# PAR_2_ Serves an Indispensable Role in Controlling PAR_4_ Oncogenicity: The β-Catenin–p53 Axis

**DOI:** 10.3390/ijms26062780

**Published:** 2025-03-19

**Authors:** Priyanga Appasamy, Jeetendra Kumar Nag, Hodaya Malka, Rachel Bar-Shavit

**Affiliations:** Sharett Institute of Oncology, Hadassah-Hebrew University Medical Center, Jerusalem 91120, Israel; priyanga.appasamy@mail.huji.ac.il (P.A.);

**Keywords:** colon cancer, G-protein coupled receptors (GPCRs), protease, protease-activated receptors (PARs), *Par2*/*f2rl1*, *Par4*/*f2rl3*, therapeutic means

## Abstract

Although the role of G-protein-coupled receptors (GPCRs) in cancer is acknowledged, GPCR-based cancer therapy is rare. Mammalian protease-activated receptors (PARs), a sub-group of GPCRs, comprise four family members, termed PAR_1–4_. Here, we demonstrate that PAR_2_ is dominant over PAR_4_ oncogene in cancer. We performed a knockdown of *Par2*/*f2rl1* and expressed C-terminally truncated PAR_2_ (TrPAR_2_), incapable of inducing signaling, to assess the impact of PAR_2_ on PAR_4_ oncogenic function by β-catenin stabilization assessment, immunoprecipitation, and xenograft tumor generation in *Nude*/*Nude* mice. PAR_2_ and PAR_4_ act together to promote tumor generation. Knockdown *Par2* and TrPAR_2_ inhibited the PAR_2_ and PAR_4_-induced β-catenin levels, nuclear dishevelled 1(DVL1), and TOP*flash* reporter activity. Likewise, PAR_2_ and PAR_4_-induced invasion and migration were inhibited when *Par2* was knocked down or in the presence of TrPAR_2_. PAR cyclic (4-4) [P*c*(4-4)], a PAR-based compound directed toward the PAR pleckstrin homology (PH)-binding site, effectively inhibited PAR_2_ oncogenic activity. P*c*(4-4) inhibition is mediated via the increase in p53 level and the up-regulation of p21 as caspase-3 as well. Overall, we showed that in the absence of PAR_2_ signaling, the PAR_4_ pro-tumor functions are significantly inhibited. P*c*(4-4) inhibits PAR_2_ acting via the modification of *wt* p53, thus offering a powerful drug measure for fighting cancer.

## 1. Introduction

Despite an increasing understanding of G-protein-coupled receptor (GPCR)-facilitated cancer pathogenesis, little is known about the role of GPCRs in epithelial malignancies. GPCRs comprise a diverse super-family of proteins, which serve as biological targets for pharmaceutical drug design. GPCR-targeted drugs presently represent nearly 30% of all therapeutics directed against a wide range of pathologies. Yet, nearly no GPCR-based drugs are clinically used in cancer [1,2,3].

In the United States (US) alone during 2024, over 2 million (e.g., 2,001,140) new cancer cases were diagnosed and over 600,000 (e.g., 611,720) cancer deaths were reported [4,5]. In fact, the 5-year survival rate for cancer in general has increased from 49% through the mid 1970s to 69% in 2013–2019. This achievement is mainly due to earlier detection, reduced smoking, and the advanced treatments, resulting in over 4 million deaths prevented since 1991.

Frizzled (FZD) receptors, a sub-group of GPCRs, are activated by Wnt ligands to initiate the canonical Wnt/β-catenin pathway. The Wnt/β-catenin signaling pathway regulates embryonic development, tissue homeostasis, and cancer [6]. Once bound to a complex comprising FZD and low-density lipoprotein receptor protein (LRP)5/6 coreceptors, Wnts initiate the β-catenin signaling pathway. This leads to the detachment of β-catenin from its cellular degradation complex, promoting β-catenin stabilization and nuclear translocation, where β-catenin acts as a transcription factor, inducing the level of a specific gene signature. As part of this process, Disheveled (DVL), a cytoplasmic adaptor protein that connects FZD to downstream components, enters the nucleus and becomes part of the transcription complex. A negative layer of regulation is provided by both RNF43 and ZNRF3, ubiquitin ligases that stimulate the degradation of FZD and LRP5/6 co-receptors. As a result of such degradation, membrane receptor availability and downstream β-catenin signaling are markedly reduced [7,8].

Mammalian protease-activated receptors (PARs) correspond to another sub-group of GPCRs and comprise four family members (PAR_1–4_), all of which are uniquely activated via proteases [9]. Proteases residing within the tumor microenvironment are either immobilized to the extracellular basement membranes as a depot storage site or, when found in a soluble form, are involved in the activation of PARs, contributing to the maintenance of tumor growth and progression. PAR_1,3,4_ are thrombin receptors and PAR_2_ is a trypsin receptor. Noticeably, PAR_2_ and PAR_4_ can be activated by the same protease. MAPkinase signaling is also involved in PAR-induced tumor growth and progression [9]. We have previously demonstrated that PAR members potently induce β-catenin stabilization, leading to β-catenin nuclear translocation and transcriptional activity [10,11,12,13]. In accordance, we have recently shown that the E3 ubiquitin ligase RNF43 negatively regulates PAR_2_ cell-surface levels and consequently, PAR_2_-induced β-catenin signaling, similar to the actions of RNF43 on FZDs. Likewise, PAR_2_ degradation is rescued by RSPO-LGR5 axis [14]. Hence, it is proposed that PARs GPCRs play a role in the β-catenin stabilization cell signaling.

Advanced bioinformatics tools and DNA sequencing have enabled the characterization of the tumor gene landscape. This has allowed for high-throughput RNA sequence analyses of selected GPCR transcriptional profiles, revealing the expression of 195 GPCRs upon cell reprogramming, leading to cancer-stem-cell (CSC) sphere formation. It was shown that PAR_4_ (*f2rl3*) and PAR_2_ (*f2rl1*) are considerably up-regulated upon CSC sphere formation [15]. In other studies, PAR_4_ has emerged as a potent oncogene that is over-expressed in cancer epithelial cells and capable of inducing tumors in vivo [16,17,18,19,20]. We have previously demonstrated that PAR_4_ is a potent oncogene capable of inducing tumors in a xenograft mouse model, in vivo [21]. In addition, we have shown earlier that P*c*(4-4), a lead backbone cyclic peptide [22,23,24] selected out of a cyclic peptide library directed toward PAR_2&4_ pleckstrin homology (PH)- binding motifs [21,25,26], effectively inhibited tumor growth.

Hierarchy exists within the PAR family. We previously demonstrated that PAR_1_-promoted cancer processes require the presence of PAR_2_. This was shown using either a *shPar2* knockdown of PAR_2_ expression or by the use of a truncated form of PAR_2_ lacking the entire cytoplasmic tail, a variant incapable of promoting cell signaling [27]. This concurs with studies by Sevigny et al. [28], demonstrating that PAR_2_ affects PAR_1_ function in neointimal arterial thickening of smooth muscle cell (SMC) growth. It also supports previous work from this group and others on PAR_1_ and PAR_2_ trans-activation [29,30]. We now ask what the inter-relations between PAR_2_ and PAR_4_ in cancer are.

Indications from various tumor models propose that the Wnt signaling and p53 pathways collaborate to promote tumor growth and progression [31,32]. Indeed, there is a cross-talk between β-catenin and p53 in cancer. Data based on genetic analysis of colorectal cancer (CRC) patients showed induced β-catenin stabilization as a result of mutated APC and β-catenin, together with a high incidence of p53 mutations [33]. In mice carrying mutant p53 (p53^R270H^), increased intestinal tumor growth and a rise in invasiveness were observed [34]. Moreover, the transformation of normal colonic stem cells via the accumulation of mutated APC, KRAS, and SMAD4 genes failed to metastasize in the presence of *wt* p53, as occurs in CRC [35,36]. At the same time, the full malignant repertoire of invasive CRC is obtained in the presence of mutated p53^R270H^ [34]. In lung cancer, for example, p53 mutations impact personalized therapy toward epidermal growth factor receptor (EGFR) mutants [37,38].

Indeed, only upon the *wt* p53 pathway does de-regulated β-catenin fully manifest its oncogenic properties. This also occurs reciprocally, whereby increased levels of *wt* p53 inhibit de-regulated β-catenin in a variety of cell settings. Therefore, a negative feedback loop links *wt* p53 and β-catenin, with an interruption of this loop most likely affecting oncogenic β-catenin tumorigenesis [39].

In the present study, our overall aim was to examine functional interactions of PAR_2_ and PAR_4_ in colon cancer. This was assessed via PAR-induced β-catenin-signaling events, PAR-PH-Akt association, colony formation, Matrigel invasion in vitro, and tumor development in vivo. In the presence of either knockdown *Par2* or truncated (Tr) PAR_2_ lacking the cytoplasmic tail, a marked inhibition of PAR_2&4_ signaling events was observed. This indicates the essential role of PAR_2_ signaling in PAR_4_-induced tumor growth. Furthermore, a P*c*(4-4) compound directed toward the PAR PH-binding motif [21,25] was shown to act through an increase in the level of *wt* p53. This highlights P*c*(4-4) as a promising anti-cancer drug.

## 2. Results

### 2.1. Knockdown of Par2/f2rl1 Inhibits Events in PAR_2_ and PAR_4_-Induced β-Catenin Stabilization and Cell Invasion

**Figure 1:**
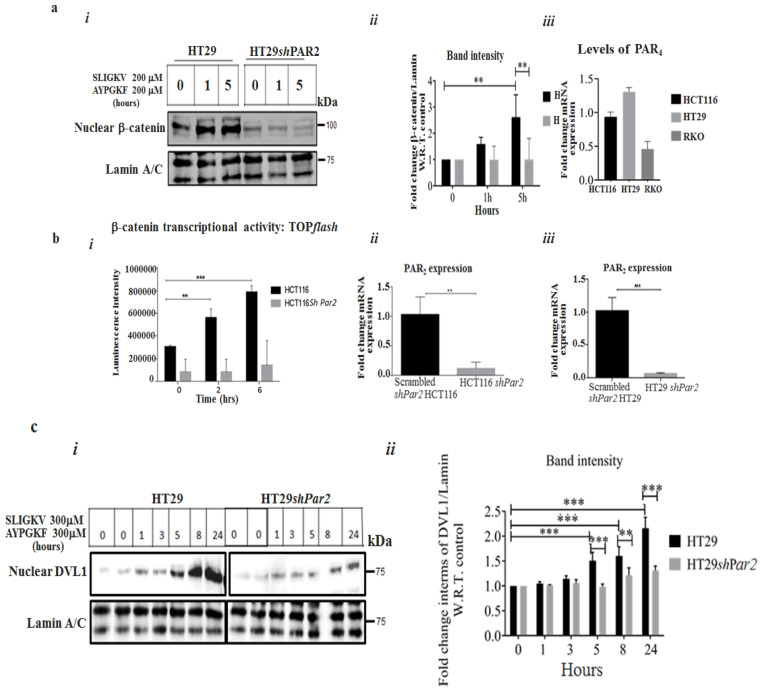
The knockdown of *Par2* inhibits PAR_2_ and PAR_4_-induced signaling. (**a**) (***i***) The knockdown of *Par2* attenuates PAR_2_ and PAR_4_-induced nuclear β-catenin levels. Western blot analysis of HT29 and HT29 *shPar2* cells. HT29 cells were infected with the lentiviral small hairpin RNA (*shPar2*) vector. The cells were activated by an addition of peptide SLIGKV and AYPGKF for the indicated intervals. Nuclear fractions were prepared and Western blot analysis was carried out for the detection of β-catenin levels in the cell nucleus. Lamin A/C served as a control for nuclear protein loading. (**a**) (***ii***) Band intensities of β-catenin/Lamin A/C. (**a**) (***iii***) qRT-PCR analyses showing levels of *Par4* in colon-cancer-cell lines. (**b**) (***i***–***iii***) TOP*flash* luciferase transcription activity in the presence of PAR_2_ and PAR_4_ HCT116 cells when *sh*RNA *Par2*/*f2RL1* is silenced. Lymphoid enhancer factor/t-cell factor (Lef/Tcf) reporter activity of PAR_2_ was markedly induced following SLIGKV and AYPGKF peptide-mediated PAR_2_ and PAR_4_ activation in HCT116 cells. Attenuated *Lef*/*Tcf* activity was obtained following PAR_2_ and PAR_4_ activation when *Par2* was silenced. Data are expressed as means ± SD. *** *p* < 0.001,** *p* < 0.003. (**c**) (***i***) The knockdown of *Par2* attenuates PAR_2&4_-induced nuclear levels of DVL1. Western blot analysis of HT29 and HT29 *sh*-*Par2* cells. Cells were activated by an addition of the SLIGKV and AYPGKF peptides for the indicated intervals. Nuclear fractions were obtained and Western blot analysis was carried out for detection of DVL1 by anti-DVL-1 antibodies. Lamin A/C served as a control for nuclear protein loading. (**c**) (***ii***) Band intensities of DVL1/Lamin A/C. A representative of three independent experiments is shown.

Upon the knockdown of *Par2* alone, the inhibition of the induced β-catenin level signaling was obtained (Figure 1a(*i*),a(*ii*)). High expression levels of both *Par2*/*f2rl1* and *Par4*/*f2rl3* (in addition to other oncogenes) were observed in HCT116 and HT29 cells (Figure 1a(*iii*)). Significantly, while we previously demonstrated evidence of the potent individual activation of β-catenin stabilization [12,14,40] by PAR_2_ via the addition of SLIGKV peptide or by PAR_4_ via AYPGKF peptide, we now aimed to elucidate the relative impact of PAR_2_ on PAR_2_ and PAR_4_ function. For the assessment of the relative impact of *Par2*/*f2rl1* on pro-tumor signaling, we knocked down *Par2* using *sh*RNA-*Par2* in colon-cancer-cell lines (i.e., HCT116 and HT29). Decreased *Par2* levels were observed, compared with those in scrambled *shPar2*-infected cells (Figure 1b(*ii*),b(*iii*)). When combined, the SLIGKV and AYPGKF peptides induced the activation of PAR_2_ [12] and PAR_4_, leading to induced β-catenin stabilization. Along this line, an increased TOP*flash* reporter activity of β-catenin transcription function was obtained following PAR_2_ and PAR_4_ activation. When *Par2* was knocked down, a marked inhibition of PAR_2_ and PAR_4_-induced TOP*flash* activity was observed (Figure 1b). When levels of β-catenin were evaluated in HT29 cells, high β-catenin levels were seen following peptide SLIGKV- and AYPGKF-mediated PAR_2_ and PAR_4_ activation. Upon the silencing of *Par2* RNA levels (it is assumed that under these conditions, levels of their respective protein levels are reduced accordingly, although not measured directly) in HT29 cells, a marked inhibition of β-catenin level was seen (Figure 1a(*i*)–(*iii*)). The activation of PAR_2_ induced DVL localization into the cell nucleus. Nuclear DVL now becomes part of a transcriptional complex composed, among others, of c-Jun, β-catenin, and Tcf [41]. While abundant nuclear DVL1 levels were observed following the activation of both PAR_2_ and PAR_4_, (Figure 1c(*i*),c(*ii*)), upon *Par2* knockdown, a significant inhibition of nuclear DVL1 level was obtained. Overall, these results point to the essential requirement of PAR_2_ expression for PAR_2_ and PAR_4_-induced β-catenin stabilization events.

**Figure 2:**
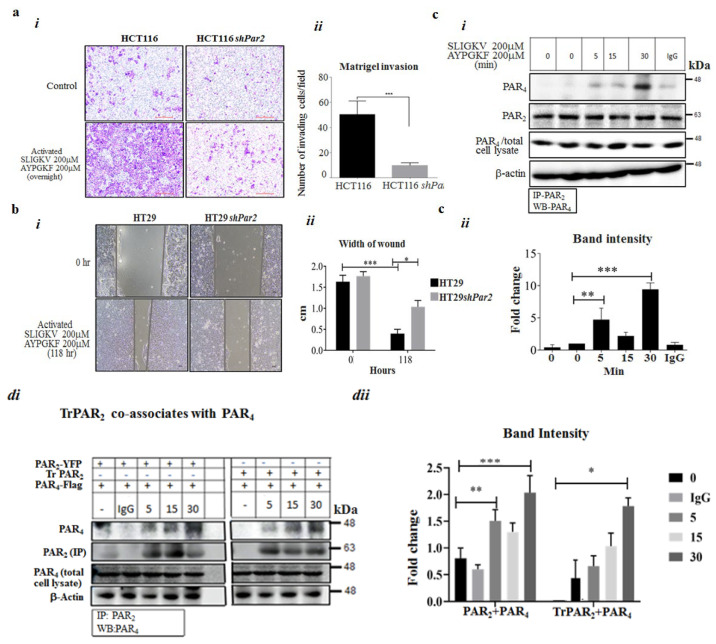
Silencing of *Par2* in HCT116 cells inhibits PAR_2_ and PAR_4_ -induced invasion. (**a**) (***i***) Matrigel invasion of *sh*RNA-*Par2*-infected HCT116 cells versus *wt* HCT116 cells. (**a**) (***ii***) Histograms represent the quantification of cell/HPF invasion of the Matrigel layer. Unpaired Student’s *t* test was used to assess the results. (**b**) (***i***) Silencing of *Par2* in HT29 cells inhibits peptide SLIGKV- and AYPGKF-induced PAR_2_ and PAR_4_ cell migration/proliferation. A wound-scratch assay in HT29 *shPar2*-infected cells and HT29 cells. A cell monolayer grown to 90% confluency was “starved” for 16 h (h) prior to performing the scratch. Peptide SLIGKV- and AYPGKF-mediated activation of PAR_2_ and PAR_4_ was carried out for 118 h. (**b**) (***ii***) Histograms represent the quantification of the space measured (cm) after 118 h. Unpaired Student’s *t* test was used for data comparison. (**c**) (***i***) Co-association of PAR_2_ and PAR_4_ shown by co-immunoprecipitation. HEK293 cells were transiently transfected with the *Par2*/*f2rl1* and *Par4*/*f2rl3* plasmids and treated with the SLIGKV and AYPGKF peptides for the indicated intervals (min). Note that 0 indicates time prior to PARs activation for 15 and 30 min. Since the association between PAR_2_ and PAR_4_ is determined relative to the time prior to activation (e.g., 0), we performed it twice. Cell lysates were then prepared and immunoprecipitated using anti-PAR_2_ antibodies or IgG. (**c**) (***ii***) Evaluation of band intensities; PAR_4_ per PAR_2_ levels. A representative of the three independent experiments performed is shown. (**d**) (***i***) TrPAR_2_ is associated with PAR_4_ and co-immunoprecipitation. HEK293 cells were transiently transfected with the *flag*-*Par4*, *tr*-*Par2*, and *wtYFP*-*Par2* plasmids. The activation of both PAR_2_ and PAR_4_ by the SLIGKV and AYPGKF peptides was carried out for the indicated intervals (min). Cell lysates were immunoprecipitated using anti-PAR_2_ antibodies or IgG, and the PAR_4_ level was detected by Western blot. The levels of PAR_2_ and PAR_4_ are shown as controls for the IP analysis. (**d**) (***ii***) The band intensity of immunoprecipitated PAR_4_/PAR_2_ is shown. These are representative of three independent experiments. *** *p* < 0.001; ** *p* < 0.01; * *p* < 0.05.

The activation of PAR_4_ and PAR_2_ enhanced the invasion of HCT116 cells through Matrigel-coated filter membranes. This stands in contrast to the marked inhibition of PAR_2_ and PAR_4_-induced invasion seen when *Par2* was knocked down (Figure 2a). Similarly, PAR_2_ and PAR_4_ activation resulted in a covering of the space generated in a wound-scratch assay (Figure 2b). When *Par2* was silenced, cell proliferation/migration in the assay was potently inhibited.

### 2.2. Co-Association of PAR_2_ and PAR_4_

It was suggested that PAR_2_ and PAR_4_ are localized in proximity, thereby enabling their action as a single functional unit upon activation. To obtain direct evidence for PAR_2_–PAR_4_ heterodimer formation, we performed co-immunoprecipitation (co-IP) analysis. For this purpose, *wt Par2* and *wt Par4* were ectopically over-expressed in HEK293 cells. The cells were treated with both the SLIGKV and AYPGKF peptides for various intervals and were further processed to obtain cell lysates. Next, IP was carried out using anti-PAR_2_ antibodies, with IgG serving as a control. As shown, co-association between PAR_2_ and PAR_4_ was observed after 5 and 10 min, reaching maximal association after 30 min of activation of both receptors (Figure 2c). This result supports our notion that PAR_2_ acts in conjunction with PAR_4_, forming a PAR_2_–PAR_4_ complex that can be observed as soon as 5 min after activation and for up to 30 min.

Similarly, a truncated form of PAR_2_, TrPAR_2_, lacking the cytoplasmic tail, is capable of co-associating with PAR_4_, as shown by the co-IP-based capture of PAR_4_ and TrPAR_2_ (Figure 2d(*i*),d(*ii*)). Co-association was obtained after the transient transfection of HEK293 cells with plasmids containing *Par4*/*f2rl3* and *TrPar2*/*f2rl1*, as compared with transfection with plasmid containing both *wt Par4*/*f2rl3* and *wt Par2*/*f2rl1*. Maximal co-association was seen after 30 min of activation of both PAR_2&4_ following activation induced by the AYPGKF and SLIGKV peptides.

### 2.3. TrPAR_2_ Inhibits PAR_2&4_-Induced β-Catenin Stabilization, Transcriptional Activity, Colony Formation and Stem-Cell Marker Levels

**Figure 3:**
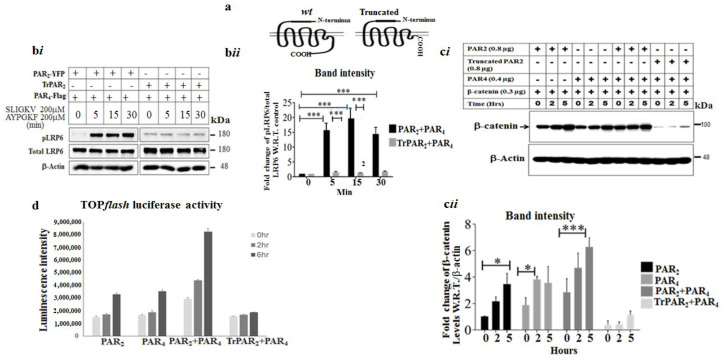
TrPAR_2_ inhibits events in PAR_2_ and PAR_4_-induced β-catenin stabilization. (**a**) A schematic representation of PAR_2_ and TrPAR_2_. (**b**) (***i***) PAR_2_ and PAR_4_ -induced phospho (p)LRP6 in the presence or absence of Tr*Par2*. HEK293 cells were transiently transfected with the *Par2*, *Par4*, and *flag*-β-catenin plasmids. The activation of both receptors by the SLIGKV and AYPGKF peptides was carried out for the indicated intervals (min). Western blot was carried out to detect pLRP6 and total LRP6. β-actin served as a control for protein loading. (**b**) (***ii***) The evaluation of band intensities; pLRP6 versus LRP6. A representative of three independent experiments performed is shown. (**c**) (***i***) PAR_2_ and PAR_4_-induced β-catenin stabilization in the presence or absence of Tr*Par2*. Levels of β-catenin were increased following SLIGKV and AYPGKF peptide-mediated activation of PAR_2_ and/or PAR_4_. This was shown in HEK293 cells following transient transfection with the *Par2*, *Par4*, and *flag*-β-catenin plasmids. In contrast, no enhancement of PAR_2_ and PAR_4_-induced β-catenin stabilization was observed when TrPAR_2_ was present. β-actin served as a control for protein loading. (**c**) (***ii***) Evaluation of band intensities; β-catenin versus β-actin. (**d**) Lef/Tcf transcriptional activity by PAR_2_ and PAR_4_. The transiently transfected HEK293 cells as described for panel c were used. The activation of PAR_2_ and/or PAR_4_ induced Lef/Tcf *Par2* and *Par4* (TOPflash activity) transcriptional activity, in the presence of either *wt* PAR_2_ and PAR_4_ or TrPAR_2_ and PAR_4_. Data are expressed as means ± SD. *** *p* < 0.001, * *p* < 0.05. A representative of three independent experiments is shown. The figures shown are representative of three independent experiments.

We next considered the effect of TrPAR_2_ on PAR_2_ and PAR_4_-induced tumor events in vitro and in vivo. The presence of a TrPAR_2_ lacking the cytoplasmic tail (Figure 3a), and hence incapable of promoting signaling, stressed the significance of PAR_2_-induced signaling events in PAR_4_ and PAR_2_-mediated activities. We transfected HEK293 cells with the *TrPar2*/*f2rl1* and *wt Par4*/*f2rl3* plasmids, as well as *flg*-β-catenin constructs, and compared their functions with that of cells transfected with plasmids for *wt Par2*/*f2rl1*, *Par4*/*f2rl3*, and *flg*-β-catenin, whereas TrPAR_2_ was well expressed on the cell-surface membrane, as we previously reported [27], and a marked inhibition of LRP6 phosphorylation was seen. In contrast, robust LRP6 phosphorylation was observed following the activation of both PAR_2_ and PAR_4_ (Figure 3b(*i*),b(*ii*)). In addition to inhibiting pLRP6 levels, TrPAR_2_ also inhibited β-catenin levels. At the same time, a powerful enhancement of β-catenin levels was obtained following the transfection and activation of *Par2*/*f2rl1* and/or *Par4*/*f2rl3* either individually, or both. These enhanced β-catenin levels are potently inhibited in the presence of TrPAR_2_ (Figure 3c(*i*),c(*ii*)). Similarly, PAR-induced TOP*flash* β-catenin reporter activity observed following the activation of PAR_2_ and/or PAR_4_ is indicative of PAR-induced β-catenin transcriptional activity. In the presence of TrPAR_2_, the powerful inhibition of both PAR_2_- and PAR_4_-induced TOP*flash* activity was obtained (Figure 3d).

**Figure 4:**
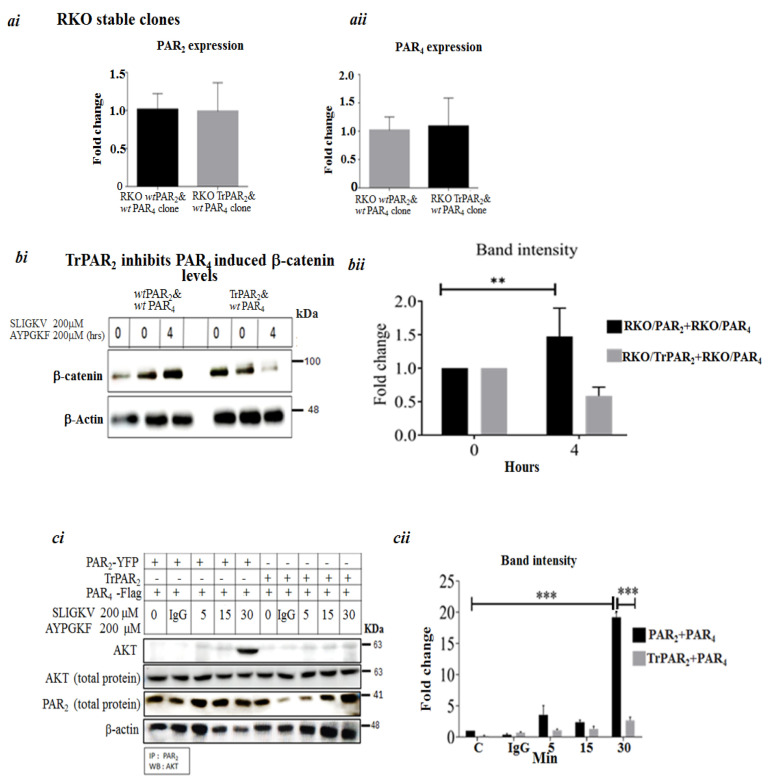
TrPAR_2_ inhibits PAR_2_ and PAR_4_-induced β-catenin protein and PAR_2_ and PAR_4_ -PH-Akt association. (**a**) (***i***) The characterization of RKO stable clones. qRT-PCR analysis. The level of PAR_2_ expression is shown. (**a**) (***ii***) RT-qPCR analysis. The level of PAR_4_ expression is shown. (**b**) (***i***) PAR_2_ and PAR_4_-induced β-catenin protein levels in the stable RKO clones. Levels of β-catenin following SLIGKV and AYPGKF peptide-mediated activation of PAR_2_ and PAR_4_ are shown. This was carried out in the presence of either *wt* PAR_2_ and PAR_4_ or TrPAR_2_ and PAR_4_. Note that 0 indicates the time prior to PAR activation, and 4 stands for 4 h activation. Since the increased level of β-catenin following activation is determined relative to the time prior to activation (e.g., zero time) we performed it twice. GAPDH serves as a control for protein loading. (**b**) (***ii***) The band intensity of the Western blot shown in b*i* is presented. (**c**) (***i***) TrPAR_2_ inhibits peptide SLIGKV- and AYPGKF-induced PAR_2_ and PAR_4_ -PH-Akt association. HEK 293 cells were transiently transfected with either plasmid pairs *wtPar2* and *wtPar4* or *trPar2* and *wtPar4*. Peptide SLIGKV- and AYPGKF-mediated activation was performed for the indicated intervals (min). Cell lysates were immunoprecipitated with anti-PAR_2_ and anti-PAR_4_ antibodies and Western blotted for the detection of Akt. Levels of AKT, PAR_2_, and PAR_4_ in total cell lysates are shown as controls. (**c**) (***ii***) The evaluation of band intensities; Akt versus PAR_2_ levels. A representative of three independent experiments performed is shown. Data were expressed as mean ± SD. *** *p* < 0.001 (highest significance) or mean ± SD. ** *p* < 0.001. The figures shown are representative of three independent experiments.

Next, we generated stable clones of RKO cells, a poorly differentiated colon-cancer-cell line, transformed on the background of mismatch repair. The clones generated over-expressed *wt Par2*/*f2rl1* and *wt Par4*/*f2rl3*, separately and in combination of both PAR_2_ and PAR_4_, as well as *TrPar2*/*f2rl1* and *wt Par4*/*f2rl3* (Figure 4a). PAR-induced levels of β-catenin stabilization were then assessed in these clones.

While increased β-catenin levels were seen following SLIGKV and AYPGKF peptide-mediated activation of *wt* over-expressing clones, β-catenin levels were attenuated in clones over-expressing TrPAR_2_ and *wt* PAR_4_ (Figure 4b).

Previously, we identified PH-binding motifs within the PAR_2_ and PAR_4_ C-terminal tails that associate with PH-containing signal proteins, thus providing a powerful platform for drug design [21,25]. We now demonstrate that in the presence of *TrPar2*/*f2rl1*, the association between PAR and PH-Akt seen in both *wt* PAR_2_ and *wt* PAR_4_ was potently inhibited (Figure 4c). Such inhibition takes place despite *TrPar2* being well expressed, as shown previously [27]. Together, these data support the conclusion that PAR_2_ signaling is required for PAR_4_ function.

**Figure 5:**
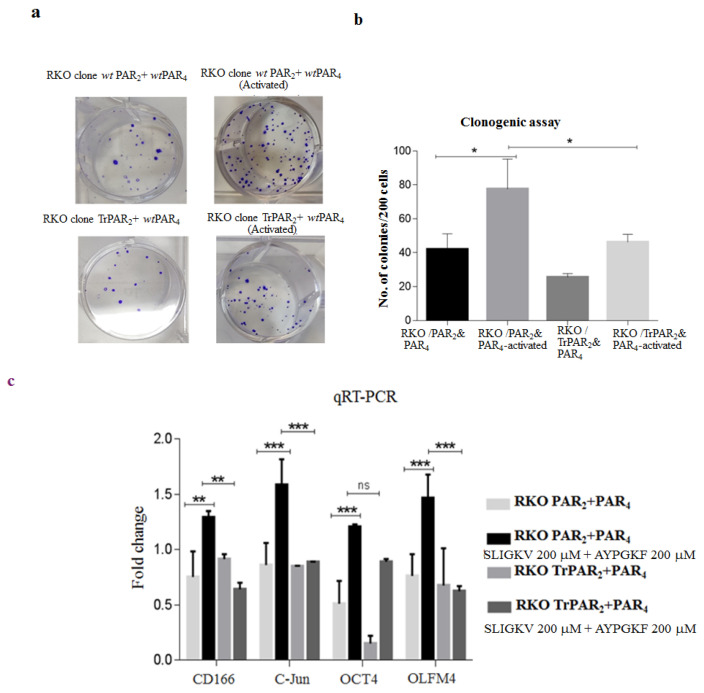
TrPAR_2_ inhibits PAR_2_ and PAR_4_-induced colony formation and expression of stem-cell markers. (**a**) Colony formation: The colony formation of *wt* PAR_2_ and *wt* PAR_4_ cells, as compared to *tr* PAR_2_ and *wt* PAR_4_ stable RKO clones. While abundant colonies were observed in *wt* PAR_2&4_-over-expressing clones, marked inhibition was seen when clones over-expressing TrPAR_2_ and *wt* PAR_4_ were evaluated. (**b**) Histograms representing the number of colonies shown in (**a**). The number of colonies shown in panel (**a**) is presented via histograms. Note that 0 indicates time prior to PAR activation, and 4 stands for 4 h activation. (**c**) Expression of the stem-cell markers CD166, cJUN, OCT4 and OLFM4 were measured by qRT-PCR. Data show means ± SD, * = *p* ≤ 0.05; ** = *p* ≤ 0.01; *** = *p* ≤ 0.001. The experiment was performed three times. ns: not significant.

A colony formation assay demonstrated the generation of abundant colonies upon activation of both PAR receptors. A marked inhibition of colony-forming ability was observed in the presence of the TrPAR_2_ variant and *wt* PAR_4_ (Figure 5a,b). Furthermore, elevated levels of stem-cell markers were seen upon activation of both PAR_2_ and PAR_4_ (Figure 5c). Reduced levels of these markers were obtained in the presence of TrPAR_2_ and *wt* PAR_4_ (Figure 5c). It is assumed that the levels of their proteins were inhibited accordingly, although not measured directly. Hence, the oncogenic activity of PAR_4_ is markedly inhibited when PAR_2_ is incapable of initiating cell signaling.

### 2.4. TrPAR_2_ Inhibits PAR_2_ and PAR_4_-Induced Tumor Growth In Vivo

**Figure 6:**
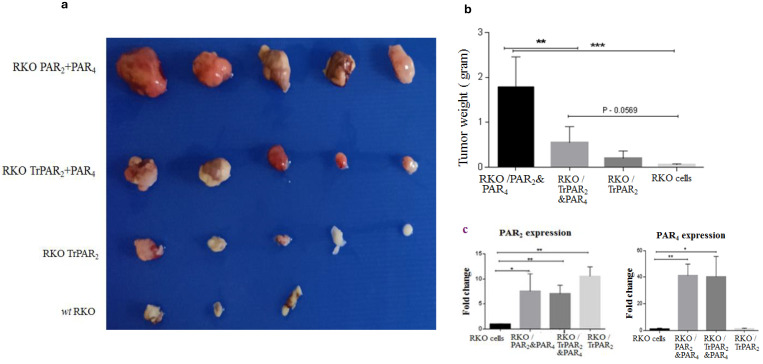
TrPAR_2_ inhibits PAR_2_ and PAR_4_-induced tumors. (**a**). Stable RKO clones: RKO/*wt Par2* and *wt Par4*; tr*Par2* and *wt Par4*; *trPar2* and *wt* RKO cells (1 × 10^6^ cells) were inoculated subcutaneously into nude mice. The mice were sacrificed after 35 days. (**b**). The tumor weight of each of the inoculated clones is shown. (**c**). Levels of PAR_2_ and PAR_4_ expression were determined by qRT-PCR in the mice inoculated with the clones. Data were expressed as mean ± SD. *** *p* < 0.001 (highest significance) or mean ± SD. ** *p* < 0.001. One of three independent experiments is shown. * *p* < 0.05.

To elucidate the dominant role of PAR_2_ over PAR_4_ in xenograft tumor growth in vivo, the following approach was taken. Stable RKO clones were inoculated subcutaneously into nude mice. The mice were monitored every other day for 35 days (or until the tumors became clearly noticeable), sacrificed, and the tumors were excised and embedded in paraffin. Whereas large tumors (e.g., ~1.2 cm) were observed in mice inoculated with the RKO clones over-expressing both *wt* PAR_2_ and *wt* PAR_4_, only a few to almost no tumors were seen following the injection of clones expressing TrPAR_2_ and *wt* PAR_4_. Likewise, only very small tumors were obtained when clones expressing either Tr*Par2* alone or *wt* RKO cells were inoculated into mice (Figure 6). The inhibition obtained was 4.5-fold higher for TrPAR_2_ and *wt* PAR_4_-generated tumors, as compared with *wt* PAR_2&4_-generated tumors. Together, both the in vitro and in vivo data obtained in the presence of a TrPAR_2_ indicated that TrPAR_2_ has a negative inhibitory effect on PAR_4_ and PAR_2_-induced tumor-promoting processes, similar to the inhibition seen following *sh*RNA-mediated *TrPar2*/*f2rl1* gene silencing.

### 2.5. The Oncogenic Properties of PAR_2_ Are Significantly Inhibited in the Presence of Pc(4-4) via Induced wt p53 Levels

We previously selected a P*c*(4-4) compound from a library of cyclic peptides directed against the PAR_2_/or PAR_4_ PH-binding motif [21]. As we have shown, P*c*(4-4) potently inhibited PAR-induced Akt association, cell migration, and invasion in vitro, as well as tumor generation in vivo [21,25]. PAR_2_ activity is necessary for PAR_4_ function (and also for PAR_1_; 27).

Now, we continue our assessment of the molecular mechanism of PAR_2_ by using P*c*(4-4) as a potent inhibitor.

**Figure 7:**
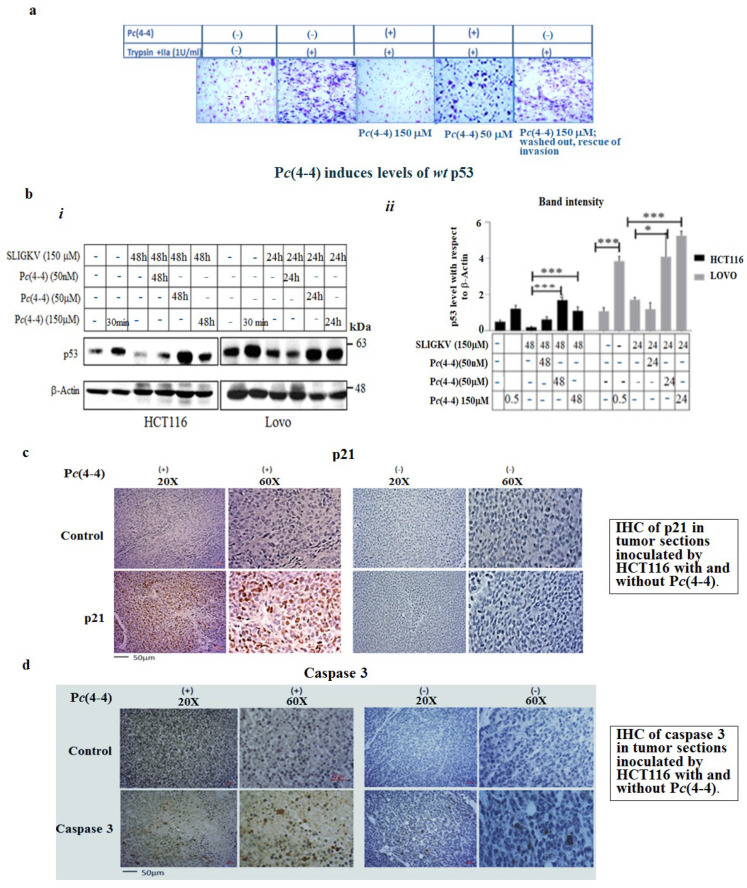
Application of P*c*(4-4) to peptide SLIGKV-activated cancer-cell lines protects levels of *wt* p53. (**a**) Matrigel invasion of protease-activated PAR_2_ and PAR_4_. HCT116 colorectal cells were treated with both trypsin and IIa (thrombin) at 1 U/mL. Cells invading Matrigel layer were obtained in the presence and absence of Pc(4-4). Rescue of invasion was obtained following the wash out of the highest P*c*(4-4) concentration. (**b**) (***i***) Western blots. Either HCT116 or Lovo cancer cells were PAR_2_-activated by the SLIGKV peptide for the indicated intervals. This was performed in the presence or absence of varying concentrations of P*c*(4-4). Following PAR_2_ activation, *wt* p53 was degraded over time, and increased levels of *wt* p53 were seen upon application of P*c*(4-4). β-actin served as a control. (**b**) (***ii***) An evaluation of the bands; levels of p53 versus β-actin. (**c**) IHC of tissue sections generated from inoculated HCT116 cells presenting high PAR_2&4_ levels [19], in the presence or absence of P*c*(4-4) treatment. Levels of p21 in the presence and absence of P*c*(4-4) in tumor-tissue sections are shown (**d**) Levels of active caspase-3 in sections of that were either treated with P*c*(4-4) or not. The figures shown are representative of three independent experiments. *** *p* < 0.001; * *p* < 0.05.

In order to assess the impact of Pc(4-4) on PAR induced tumorigenity that mimics physiological conditions by proteases activation, we performed a Matrigel invasion assay. It is shown that P*c*(4-4) potently inhibits the Matrigel invasion of trypsin and thrombin (e.g., IIa)-activated PAR_2_ and PAR_4_, in HCT116 colon cancer cells (expressing both PAR_2_ and PAR_4_). The inhibition is observed between 50 nM and 150 μM of P*c*(4-4). This inhibition is reversible, since upon extensive wash-out, increased Matrigel invasion is seen (Figure 7a).

We found that in the presence of P*c*(4-4), a significant increase in the level of *wt* p53 was observed. As shown in Figure 7b, the level of *wt* p53 in aggressive colon-cancer-cell lines (e.g., HCT116 and Lovo) were rapidly down-regulated following PAR_2_ activation. In contrast, in the presence of P*c*(4-4), sustained high levels of *wt* p53 were seen. This was shown for the lowest concentration of P*c*(4-4) tested (50 nM), with *wt* p53 levels remaining elevated for 24 and 48 h, compared to untreated cells.

Likewise, immunohistochemistry (IHC) analysis of tumor sections generated in HCT116 cells presenting high PAR_2_ and PAR_4_ levels showed increased expression of p21 upon P*c*(4-4) treatment; this was not seen in non-treated tumor sections. Similarly, increased levels of caspase-3 were observed, indicative of small tumors, as compared to the low caspase-3 levels seen in non-treated large tumor-tissue sections (Figure 7c,d).

## 3. Discussion

In this study, we demonstrated that PAR_2_ affects PAR_4_ oncogenic function. PAR_4_ requires PAR_2_ signaling for its pro-tumor roles. We showed that PAR_2_ co-localizes with PAR_4_ and acts as a single functional unit in promoting events in the β-catenin stabilization pathway and PAR-PH-Akt association. When *Par2*/*f2rl1* is knocked down, the potent inhibition of β-catenin levels, β-catenin transcriptional activity, as reported by TOP*flash*, and increased nuclear DVL1 levels were obtained. Likewise, when *Par2*/*f2rl1* was silenced, a powerful inhibition of PAR_2_ and PAR_4_-induced Matrigel invasion and cell migration/proliferation were seen. These observations are in contrast to the increased pro-tumor events induced by high PAR_2_ and PAR_4_ levels outlined above. TrPAR_2_, which is incapable of initiating cell signaling, greatly inhibited PAR_2_ and PAR_4_-induced β-catenin stabilization, TOP*flash*-reported transcriptional activity, Akt-PAR_4_ association, colony formation, and PAR_4_-induced stem-cell marker levels. These changes took place under conditions in which TrPAR_2_ effectively co-localized with PAR_4_. The importance of PAR_2_-induced signaling for PAR_4_ function was demonstrated Via the inhibition of PAR_2_ and PAR_4_-induced tumor generation in vivo. In contrast, potent tumor growth were generated by the inoculation of clones expressing both PAR_2_ and PAR_4_. To further understand the oncogenic mechanism of action of PAR_2_, we studied the impact of P*c*(4-4), a PAR_2_ inhibitor directed to PAR_2_ and PAR_4_ PH-binding motifs. We demonstrated that upon P*c*(4-4) treatment, increased levels of p53 were observed. In parallel, P*c*(4-4) was shown to inhibit PAR-induced tumors [21]. In fact, P*c*(4-4) is intended to be a drug in the fight against cancer. We are now in the process of measuring its half-life in blood and its efficient concentration, as well as its effect on physiological protease-activated PAR_2_ and PAR_4_.

We previously demonstrated that PAR_1_ and PAR_2_ act together as a single functional unit while promoting breast cancer growth [27], as also shown by others in different settings [26,27,28]. Whether the exposed internal ligand of the PAR_2_ sequence transactivates PAR_4_ or whether the PAR_2_–PAR_4_ heterodimer is formed by another route remains to be determined. The bioinformatics assignment of PAR_4_ as an oncogene was previously reported [15]. Along with this line of evidence, we have recently shown that PAR_4_ is a potent oncogene capable of inducing tumors in vivo [21]. However, publications offering opposing roles for PAR_4_ in cancer biology exist. Some describe PAR_4_ as playing an inhibitory role in cancer [42,43,44,45]. Still, an increasing number of publications point to a role of PAR_4_ as an oncogene [17,46,47,48,49,50,51,52,53,54].

PAR_4_ associates with PAR_1_ to form heterodimers following thrombin activation. Mapping the PAR_1_–PAR_4_ heterodimer interface showed it involving four residues in the TM4 (transmembrane 4) of both PAR_1_ and PAR_4_ [55]. The mechanistic basis for PAR_1_–PAR_4_ heterodimer generation indicates that in human platelets, PAR_1_ functions as a co-factor for thrombin activation of PAR_4_. Consequently, thrombin acts as a bivalent agonist of both receptors [56,57]. Whereas PAR_4_ is required for later stages of platelet function, PAR_1_ is essential for early stages in platelet activation [58].

The fact that PAR family members work in concert and are inter-dependent is interesting but not unique. The EGF receptor (EGFR)/erbB family, comprising EGFR/erbB1/Her1, erbB2/Her2, erbB3/Her3, and erbB4/Her4, provides another such example. erbB3 lacks kinase activity; thus, it cannot induce cell proliferation. Moreover, cells expressing a mutant form of *Her2* incapable of binding EGF ligand exhibit a low rate of cell proliferation. Only cells expressing both erbB3 and mutant *Her2* receptors show strong kinase activity and robust cell proliferation upon addition of an EGF ligand [58,59,60,61]. With regard to the PAR family, PAR_2_–PAR_4_ heterodimers are functional, and as demonstrated here, PAR_2_ acts as a dominant receptor, relative to PAR_4_ and PAR_1_ [27].

In fact, PARs transactivate EGFR, in this respect we have previously demonstrated that activation of PAR_4_ for example, directly induces EGFR tyrosine (Y) phosphorylation. Application of P*c*(4-4) potently inhibits PAR-induced pY-EGFR [21].

Our findings reported here agree with previous work, demonstrating that the activation of PAR_2_ leads to the up-regulation of Bcl2L12 and the inhibition of p53 in lung cancer [58,62]. It is well known that p53 is a common denominator in the etiology of different sub-types of human cancers. Studies of p53 have contributed to our understanding of the cancerous process and provided insight into the development of tumor growth. Yet, one should keep in mind that signaling pathways linking p53-like cellular pathways should not be evaluated as an isolated element [63,64,65]. It is necessary to consider the intertwined networks into which p53-associated signaling is tightly linked. Mutant p53 is a bona fide useful biomarker impacting therapy programs such as those for EGFR [e.g., TKI (tyrosine kinase inhibitor)-EGFR] [37], the clinical regimen [38], and a useful biomarker for glioma [66]. A specific stop point in the cell-division cycle corresponds to the earliest effects of p53 expression and is seen in all mammalian cells. This arrest was mediated Via p53-induced expression of p21WAF1/CIP1, an inhibitor of cyclin-dependent kinases. The pivotal canonical activation of p53 leads to cell apoptosis [67]. The transcription of Bax, an apoptosis gene mediator, is directly activated by p53-binding sites in the gene regulatory region [68]. The multistage apoptotic process includes the release of mitochondrial proteins, like cytochrome c, and the activation of a cascade of cysteine proteases, like pro-caspase-9, which cleaves procaspase-3, leading to committed cell death [69,70]. p53 activity is often inhibited by numerous antagonistic mechanisms, the most noticeable of which is the activation of the Mdm2 E3 ligase [63,64,65].

Proteomic and transcriptomic analyses have convincingly demonstrated that the combined gene regulation of the *wt* p53 tumor suppressor and c-Myc oncogene is key in eradicating cancer stem cells [71]. Notably, p53 and c-Myc appear in many cancer networks. The robust increase in *wt* p53 levels seen upon treatment with P*c*(4-4) highlight this compound as a powerful therapeutic compound. Similarly, the activation of *wt* p53 can be achieved by inhibitors of CKIα [72,73]. Small molecules that co-target kinase inhibitors (e.g., CKIα) in acute myeloid leukemia have been shown to induce p53 levels and inhibit c-Myc oncogene concomitantly [74]. Likewise, P*c*(4-4) was found to be a potent inducer of *wt* p53 levels and its downstream effector p21, as well as caspase-3. In summary, we have demonstrated that in the absence of PAR_2_ signaling, PAR_4_ pro-tumor functions are significantly inhibited and P*c*(4-4) acts as a potent anti-tumor agent Via a significant increase in *wt* p53 levels.

## 4. Materials and Methods

### 4.1. Animal Models

Animals used in the experiments were handled in accordance with the guidelines of the Hebrew University ethics committee (AAALAC standard). The animal ethics certificate number is MD-17368-5.

### 4.2. Cell Culture

HCT-116, HT29, RKO, LOVO, and HEK293 cells were obtained from the American Type Culture Collection (Manassas, VA, USA) and grown in DMEM, supplemented with 1 mM L-glutamine, 50 µg/mL streptomycin, 50 U/mL penicillin (GIBCO-BRL, Gaithersburg, MD, USA) and 10% fetal calf serum (Biological Industries, Migdal HaEmek, Israel). These cell lines were checked for authentication by the service at the genomic center BCF biomedical core facilities, Haifa, Israel.

### 4.3. Matrigel Invasion

Blind-well chemotaxis chambers with 13 mm diameter filters were used. Matrigel (AcroBiosystems, Boston, Cambridge, MA, 02142, USA; AC-M082704) was applied per blind well (50 mg/filter) as described previously [24,26].

### 4.4. Scratch-Wound Healing

The scratch-wound healing assay was performed as described previously, with some modifications [75]. In brief, HT29 and HT29 *sh*Par2 cells (3 × 10^6^/well) were seeded in 6-well plates. The cell medium was replaced with serum-free medium overnight, and equal wound areas were introduced into the monolayer. Cells were treated with 200 µM of the synthetic peptides SLIGKV or AYPGKF (GenScript; Piscataway, NJ, USA).

### 4.5. Clonogenic Assay

RKO (200) was seeded into 24-well plates, activated for both PAR_2_ and PAR_4_, and maintained until visible colonies were observed. The colonies were fixed with glutaraldehyde (6.0%, *v*/*v*; Sigma-Aldrich-Merck, Kiryat HaMada St 15, Jerusalem, Israel), stained with crystal violet (0.5%, *w*/*v*) and counted using a stereo microscope [76].

### 4.6. Plasmids and Reagents

pBJ-FLAG-*Par4* (cat #53231), *lrp6* (cat #27242), pCMV-VSV-G (cat #8454) and pCMV-dR8.2 dvpr (cat #8455) plasmids were purchased from Addgene (Watertown, MA, USA). Human PAR_2_ (*Par2*/*f2rl1*) plasmid was kindly provided by Dr. Morley D. Hollenberg (Faculty of Medicine, University of Calgary, Calgary, AB, Canada). *flg*-β-catenin was kindly provided by Dr. Ben-Neriah (Hebrew University, Jerusalem, Israel). Preparation of a TrPAR_2_ plasmid, encoding the protein lacking the cytoplasmic tail (i.e., lacking 150 residues) was carried out as previously described [25].

### 4.7. Cell Transfections and PAR Activation

Cells grown to 70–80% confluency were transfected with 0.5–1 μg of plasmid DNA using the PEI transfection reagent (Polysciences, Warrington, PA, USA). Cells were collected 48 h after transfection and protein lysates or RNA were prepared. Two hundred µM of the synthetic peptides SLIGKV or AYPGKF (GenScript; Piscataway, NJ, USA) were used to activate PAR_2_ and PAR_4_, respectively.

### 4.8. Small Hairpin (sh)-RNA Construct Preparation and Viral Particle Generation

To prepare *sh*-RNA of different genes and thereby knockdown gene expression, the desired sequence was successfully cloned into the plentilox3.7 (pLL3.7; #11795; Addgene, Watertown, MA, USA) lentiviral vector following the protocol provided by the Addgene website (cat #11795). The target sequences were *sh*-*Par2i*: 5′-GGAAGAAGCCTTATTGGTA-3′, *sh*-*Par2ii*: 5′-GCTCTTTGT AATGTGCTTA-3, scrambled *sh*-*Par2i*: AGAGAAGTTCGAAGGTATA-3′ and scrambled *sh*-*Par2ii*: 5′-TGCTGTGATAGTTAT TCGA-3′. For the generation of viral particles, HEK293 cells were transfected with three plasmid systems that encode packaging (CMVD R8.91), envelope (CMV-VSV-G) proteins, and cloned pLL3.7 vector using PEI as transfection reagent. The medium was replaced with fresh medium 24 h later. On day three after transfection, the medium was collected, and the viral particles were concentrated 10-fold by centrifuging for 2 h at 40,000 rpm.

### 4.9. Preparation of RKO Stable Clones Expressing wt PAR_2_/wtPAR_4_ and trPAR_2_/wtPAR_4_

RKO cells (0.5 × 10^6^) were infected with *Par4* 10 × viral particles along with Polybrene infection reagent. At 72 h post transduction, the cells were subjected to puromycin selection (0.5 µg/mL). While the control cells died, transduced cells with puromycin resistance grew and were collected. These cells were then infected with *Par2* 10 × virus particles along with Polybrene. Puromycin-resistant cells were collected and either used to isolate RNA or to prepare protein lysates.

### 4.10. Quantitative Real-Time (qRT) PCR and Reverse Transcriptase (RT) PCR

RNA was extracted from cells using the GenElute RNA kit (Sigma-Aldrich, St. Louis, MO, USA). To prepare cDNA, 1 µg of RNA was reverse-transcribed using reverse transcriptase (Promega, Madison, WI, USA (Quanta bio, Beverly, MA, USA)). qRT-PCR was performed using specific forward and reverse primers for each gene analyzed. In qRT-PCR amplifications, triplicates of the 6 ng cDNA template were used with 500 nM gene-specific primers and the 2 × PerfeCTa SYBR Green mix in an RG-3000A automated rotor-gene system (Corbett Research, Sydney, Australia).

### 4.11. Cell Lysate Preparations, Immunoprecipitation (IP), and Western Blot

To prepare protein-cell lysates for IP, cells were solubilized in CelLytic M buffer (Sigma-Aldrich, Saint Louis, MO, USA). Protein-cell lysates were prepared as previously described [12,14]. Western blot analysis was performed as in previous studies [10,11,12,13,14]. In this study, experimental cell-protein extraction and lysis were carried out using RIPA (Radio-Immunoprecipitation Assay) buffer supplemented with PMSF and a protein inhibitor. Subsequently, protein concentrations were determined, and the lysates were heated with a loading buffer at 100 °C for 10 min. Thirty micrograms of proteins from each sample were loaded onto 10%. SDS-PAGE gels, transferred to PVDF membranes, blocked with 5% BSA, and then exposed to the relevant antibody. Western blots (wet Western blots) were performed. Membranes (PVDF, Thermofisher, Waltham, MA, USA; TS-88518) were blocked and probed with the appropriate primary antibodies. These are anti-FLAG (F3165; Sigma-Aldrich, Saint Louis, MO, USA), anti-β-actin (A5441; Sigma-Aldrich, Saint Louis, MO, USA), anti-β-catenin (C2206), anti-PAR_2_ (AB180953; Abcam, Cambridge, UK and SC-13,504 Santa Cruz Biotechnology, Dallas, TX, USA), anti-PAR_4_ (AB5787; Abcam (Cambridge, UK): SC-13504 Santa Cruz Biotechnology (Dallas, TX, USA)), anti-HA (901503; Biolegend, San Diego, CA, USA), anti-LRP6 (BS-7007R, Bioss Antibodies, Woburn, MA, USA), anti-phospho-LRP6 (Cell Signaling Technology, Danvers, MA, USA), anti-AKT (AB8805; Abcam, Cambridge, UK), anti-p53 (AB17990; Abcam, Cambridge, UK), anti-p21 (AB109520; Abcam, Cambridge, UK), and anti-GAPDH (AB9485; Abcam, Cambridge, UK). These antibodies were suspended in 3% BSA (#A5611, Sigma-Aldrich, Head office Kanagawa, Japan) in 10 mM Tris-HCl (T1503; Sigma-Aldrich, Saint Louis, MO, USA), pH 7.5, 100 mM NaCl, and 0.1% Tween-20 (P9416; Sigma-Aldrich, Saint Louis, MO, USA). After extensive washes, blots were incubated with secondary antibodies conjugated to horseradish peroxidase (HRP) anti-mouse (#ab6728; Abcam, Cambridge, UK) or anti-Rabbit (Abcam, Cambridge, UK; #ab6721). Immunoreactive bands were detected by the enhanced chemiluminescence (ECL) reagent (Pierce, Rockford, IL, USA).

### 4.12. TOPflash Luciferase Reporter

HEK 293 cells (0.2 × 10^6^) were seeded in 6-well plates and incubated overnight at 37 °C. The cells were transfected with desired plasmids (*wtPar2*, *trPar2* and *wtPar4)* and TOP*flash* components, as previously described [12].

### 4.13. Ectopic Tumor Xenograft Mouse Model

RKO cells (*wt* and stable clones of RKO*tr*PAR_2_, RKO*wt*PAR_2_ + *wt*PAR_4_ and RKO*tr*PAR_2_ + *wt*PAR_4_) were starved overnight, and treated the next day with the SLIGKV or AYPGKF peptides (200 µM each) for 4 h. The cells (1 × 10^6^) were injected subcutaneously into the right flank of groups of five six–eight-week-old Hsd: Athymic NudeFoxn1nu mice (nude mice). The mice were terminated when the tumor volumes reached the volume stipulated by the Animal Committee approval.

### 4.14. Immunohistochemistry (IHC)

Tumor tissue-derived paraffin-embedded slides were used for IHC, as previously described [23,25].

### 4.15. Pc(4-4)

Pc(4-4) is a cyclic peptide directed toward PAR_2_ and PAR_4_ PH-binding motifs. More detailed description appears in [21].

## 5. Conclusions

We have demonstrated that PAR_2_ is dominant over PAR_4_ in colon cancer development. The *sh*RNA silencing of *Par2*/*f2rl1*inhibits PAR_2_ and PAR_4_-induced events in the β-catenin stabilization pathway, and inhibits invasion and migration. Similarly, truncated PAR_2_ (TrPAR_2_), devoid of the cytoplasmic tail of PAR_2_, inhibits PAR_2_ and PAR_4_-induced β-catenin stabilization, PAR_4_-Akt association, stem-cell marker expression and colony formation. TrPAR_2_ inhibits xenograft PAR_2_ and PAR_4_-induced tumor growth in vivo. P*c*(4-4), a compound directed to the PAR_2_ PH-binding domain, inhibits PAR_2_ oncogenic activity Via an increase in p53 levels.

## Data Availability

Data used in this study are available upon request by the corresponding author.
